# Depletion of FoxP3^+^ Tregs improves control of larval *Echinococcus multilocularis* infection by promoting co‐stimulation and Th1/17 immunity

**DOI:** 10.1002/iid3.181

**Published:** 2017-06-16

**Authors:** Junhua Wang, Stephan Müller, Renyong Lin, Myriam Siffert, Dominique A. Vuitton, Hao Wen, Bruno Gottstein

**Affiliations:** ^1^ Vetsuisse Faculty, Department of Infectious Diseases and Pathobiology, Institute of Parasitology University of Bern Bern Switzerland; ^2^ State Key Lab Incubation Base of Xinjiang Major Diseases Research (2010DS890294) and Xinjiang Key Laboratory of Echinococcosis First Affiliated Hospital of Xinjiang Medical University Urumqi Xinjiang China; ^3^ FACSLab, c/o Institute of Pathology University of Bern Bern Switzerland; ^4^ Vetsuisse Faculty, Department of Infectious Diseases and Pathobiology, Central Animal Facilities University of Bern Bern Switzerland; ^5^ WHO‐Collaborating Centre on Prevention and Treatment of Human Echinococcosis and French National Reference Centre on Alveolar Echinococcosis University of Franche‐Comté and University Hospital Besançon France

**Keywords:** CD4^+^ CD25^+^ Treg, co‐stimulation, *Echinococcus multilocularis*, Foxp3, Th1/Th17 immunity

## Abstract

**Introduction:**

The growth potential of the tumor‐like *Echinococcus multilocularis* metacestode (causing alveolar echinococcosis, AE) is directly linked to the nature/function of the periparasitic host immune‐mediated processes. Previous studies had shown that regulatory T cells (Tregs) become gradually up‐regulated in the course of both chronic human and murine AE. Thus we now tackled the role of FoxP3^+^ Tregs and FoxP3^+^‐Treg‐regulated immune response in contributing to the control of this helminthic infection.

**Methods:**

The infection outcome in *E. multilocularis*‐infected DEREG mice was measured upon determining parasite load (wet weight of parasitic metacestode tissue). Flow cytometry and qRT‐PCR were used to assess Treg, Th17‐, Th1‐, Th2‐type immune responses and antigen presenting cell activation.

**Results:**

We showed that *E. multilocularis*‐infected DEREG‐mice treated with DT (as compared to infected control DEREG‐mice without DT application) exhibited a significantly lower parasite load, associated with a persisting capacity of co‐stimulation, and an increased Th1/Th17‐polarization.

**Conclusions:**

FoxP3^+^ Tregs appear as one of the key players in immune regulatory processes favoring (i) metacestode survival by inhibiting the maturation potential of co‐stimulatory activity and (ii) T cell exhaustion (suppressing Th1/Th17‐type immune responses). We showed as well that prospectively, targeting FoxP3^+^ Tregs could be an option to develop an immunotherapy against AE.

## Introduction

Alveolar echinococcosis (AE) is a very severe zoonotic helminthic disease, which is fatal if patients are not appropriately diagnosed and subsequently treated 1. AE is characterized by a chronically progressing hepatic damage caused by the continuous proliferation of the larval stage (metacestode) of *Echinococcus multilocularis* 2, that behaves like a slowly growing and metastasizing liver cancer, thus progressively also invading other host tissues and organs such as lungs and brain, among others 3. There is growing evidence that the composition and type of the—especially periparasitic—immune response elicited by *E. multilocularis*‐infection causatively influences the outcome and progression of disease 2, 3, ranging from resistance (self‐cure) to a rapidly evolving fatality for the patient (high susceptibility) 4. In humans, a T helper cell (Th)2‐oriented immunity is associated with increased susceptibility to disease leading to chronic AE, while Th1 cell‐activation has been linked to reduced or abrogated metacestode proliferation, or even protection, which occurs when the parasite becomes aborted, that is, resulting in the formation of “died‐out” lesions 2, 3. Experimental murine AE is immunologically characterized by an initial Th1‐oriented response during the early stage of infection (till 1 month post‐infection (p.i.)) that gradually switches to a more dominant Th2‐orientation during the chronic phase of AE (2–4 months p.i.). This still mostly mixed Th1/Th2 profile associates with the expression of pro‐inflammatory cytokines in the peri‐parasitic granuloma, and partial/relative protective immunity (restricted parasite growth) occurs through fibrosis and necrosis by potentially inflicting a starving process to the parasite 4. It has been previously reported that CD4^+^CD25^+^ T regulatory cells (Tregs) appeared quantitatively up‐regulated in human AE, and it was claimed that this up‐regulation associated to the blunting of immune responses to specific antigens, and/or to the suppression of the secretion of pro‐inflammatory cytokines, especially through high interleukin (IL)‐10 and transforming growth factor beta1 (TGF‐β1) production 5. In the experimental mouse model, increased CD4^+^CD25^+^ Tregs were also observed within the peritoneal cell population, a result that concurred with other findings demonstrating that *E. multilocularis* antigens promote T cell differentiation into Treg cells 6.

So far, only few studies have reported on the possible involvement of Tregs in the immune regulation of murine AE 4, 7, 8, none with regard to the possible mechanism of FoxP3‐regulation. The major aims of the present study were: (i) to address the role of FoxP3^+^ Tregs in T cell reactivity as well as its effect on co‐stimulation at the early (1 month p.i.) and at a late chronic (4 months p.i.) stage of *E. multilocularis* infection, employing a mouse model that allows to induce the depletion of regulatory T cells (DEREG); (ii) to explore whether FoxP3^+^ Tregs could be envisaged as an immunotherapeutical candidate for supporting treatment against AE; (iii) to provide a comprehensive picture of the possible mechanism and pathways involved in immune regulation at the early stage of *E. multilocularis* infection. To achieve these goals, we investigated the co‐stimulation status of CD11b^+^ and CD11c^+^ APCs, together with Th1/Th2‐related plus Treg/Th17‐related cytokine expression levels, at the early infection stage in an experimental model with active or depleted FoxP3‐expression.

## Results

### *E. multilocularis* infection/excretory/secretory proteins induces Treg‐related nuclear transcriptional factor and cytokine up‐regulation

**Figure 1:**
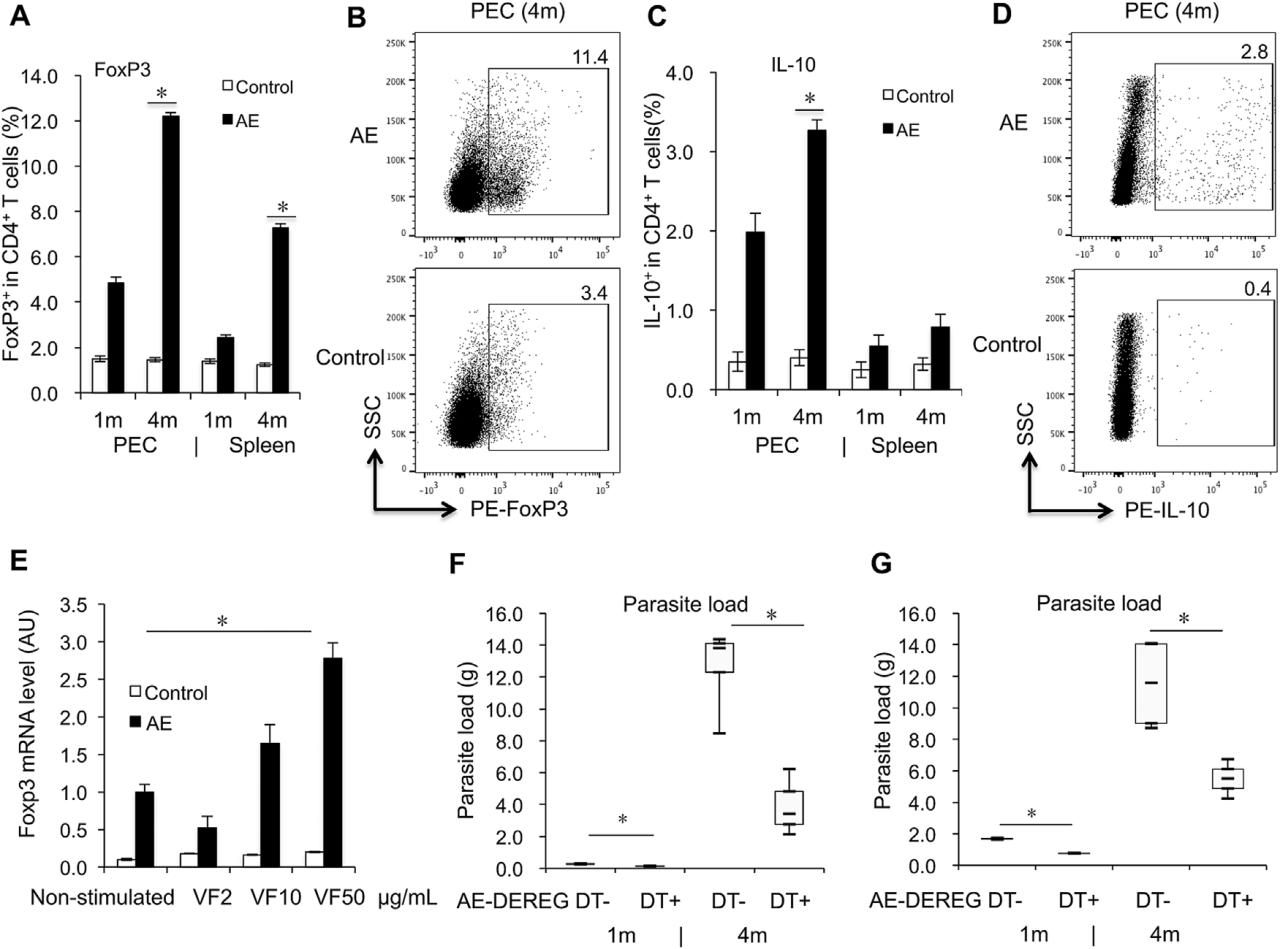
FoxP3‐ and IL‐10‐levels affected by *E. multilocularis* infection, and association between FoxP3 and metabolites, parasite load development in *E. multilocularis*‐infected mice. (A) Frequency of FoxP3^+^ T cells within CD4^+^ T cells in PECs and spleen cells from AE‐WT and Control‐WT mice at 1 month and 4 months post‐infection. (B) Representative images of FoxP3^+^ T cells within CD4^+^ T cells in PECs from both AE‐WT and Control‐WT mice at 4 months post‐infection. (C) Frequency of IL‐10^+^ T cells within CD4^+^ T cells in PECs and spleen cells from AE‐WT and Control‐WT mice at 1 month and 4 months post‐infection. (D) Representative images of IL‐10^+^ T cells within CD4^+^ T cells in PECs from both AE‐WT and Control‐WT mice at 4 months post‐infection. Comparison between groups was performed using a one‐way ANOVA with Bonferroni's multiple comparison post‐test for statistical analysis. *p < 0.006 (E) *foxp3* gene expression in spleen cells from AE‐WT and Control‐WT mice, co‐cultured with 2, 10, 50 µg/mL *E. multiloculari*vesicle fluid (VF) (measured by qRT‐PCR). The same cell reactions performed without VF were used as non‐stimulated controls. *p < 0.05. AU: arbitrary units. (F) Parasite load in AE‐DEREG DT‐ and AE‐DEREG DT+ mice assessed by wet weight measurement at 1 month and 4 months post‐ infection. DT application with 110 ng/injection/mouse started 1 day before infection and was maintained for 4 months (three times/week). (G) Parasite load in AE‐DEREG DT‐ and AE‐DEREG DT+ mice assessed by wet weight measurement at 1 month and 4 months post‐ infection. DT application with 110 ng/injection/mouse (three times/week) started 1 day before infection and was maintained for 1 month. Data represent mean ± SD of three independent experiments of a total of 8–10 mice in each group (4–5 mice per group in each independent experiment). Comparison between groups was performed using a one‐way ANOVA for statistical analysis. *p < 0.05. “DEREG DT‐,” *foxp3 inducible* knock‐down mice (DEREG mice) without DT application; “DEREG DT+,” DEREG mice with DT application; “AE‐ DEREG DT‐,” *E. multilocularis*‐infected DEREG without DT application; “AE‐ DEREG DT+,” *E. multilocularis*‐infected DEREG mice with DT application. “Control,” non‐infected mice; “1 m,” 1‐month p.i.; “4 m,” 4 months p.i. “PEC,” peritoneal exudate cells; “Spleen,” spleen cells.

FoxP3^+^ and IL‐10^+^ frequency within CD4^+^ T cells was significantly higher in peritoneal exudate cell PECs and spleen cells of *E. multilocularis* infected (AE‐WT) mice at 4 months post‐infection (p.i.) when compared to non‐infected WT‐controls (Fig. 1A–D). Overall, and with regard to those two parameters, PECs seemed to be more affected by infection than spleen cells. To further explore the effect of parasite metabolic *E. multilocularis* vesicle fluid (VF) on Tregs, spleen cells from AE‐WT mice and non‐infected WT controls were each co‐cultured with three different concentrations of VF (2 µg/mL, 10 µg/mL, 50µg/mL, respectively), and *foxp3* gene‐expression levels were subsequently determined by qRT‐PCR. Findings indicated that *foxp3* gene‐expression levels were up‐regulated in response to high concentration of VF (50 µg/mL), when compared to non‐infected animals (Fig. 1E).

### FoxP3 plays a critical role in the overall infection control

To assess the putative role of FoxP3 in the control of parasite growth/proliferation, *E. multilocularis*‐infected DEREG mice with/without DT application and corresponding control WT littermates, respectively, were analyzed after 1 and 4 months p.i. with regard to the recovered parasite mass. In a first experiment (experimental step a), DT application (110 ng/injection/mouse) was initiated one day prior to infection and was maintained during 4 months (three times/week). We found that AE‐DEREG mice with DT application(AE‐DEREG DT+) had a significantly lower parasite load when compared to AE‐DEREG mice without DT application (AE‐DEREG DT‐) both at 1‐month p.i. (0.14 ± 0.02 vs. 0.27 ± 0.03 g, *p* = 0.044) and 4 months p.i. (3.96 ± 2.09 vs. 12.62 ± 2.79 g, *p* = 0.005) (Fig. 1F). To study whether FoxP3 is involved in orientation of the immune response at the early stage of infection only (experimental step b), we applied DT (110 ng/mouse) as above but maintained DT application for only 1 month, and then let the infection proceed for another 3 months in the absence of further DT application. The parasite load was significantly lower in AE‐ DEREG DT+ mice when compared to AE‐DEREG DT‐ at 1‐month p.i. (0.15 ± 0.01 vs. 0.26 ± 0.04 g, *p* = 0.005) and still significantly lower at 4 months p.i.. (5.50 ± 0.57 vs. 12.14 ± 3.23 g, *p* = 0.027) (Fig. 1G). Respective flow cytometric analyses showed that FoxP3 was strongly suppressed after 1 month DT application, but less so after 3‐months DT‐application (Supplementary Fig. S1A–D).

### Co‐stimulation of antigen presenting cells (APCs) is less affected in DEREG DT+ than in DEREG DT‐ mice at the early stage of infection (1 month p.i.)

**Figure 2:**
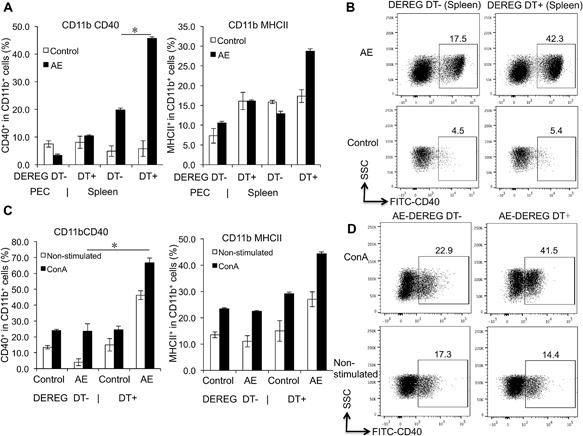
CD40 and MHCII expression levels in both CD11b^+^ APCs in both AE‐DEREG DT‐ and AE‐DEREG DT+ mice at 1 month post‐infection, and in response to ConA. (A) Frequency of co‐stimulation markers CD40 and MHCII within CD11b^+^ APCs in peritoneal and spleen cells from AE‐DEREG DT‐ and AE‐DEREG DT+ mice 1 month post infection. (B) Representative images of CD40^+^ cells within CD11b^+^ APCs in spleen cells from both AE‐DEREG DT‐ and AE‐DEREG DT+ mice at 1 month post‐infection, non‐infected mice as Control mice. (C) Frequency of co‐stimulation markers CD40 and MHCII within CD11b^+^ APCs in spleen cells from AE‐DEREG DT‐ and AE‐DEREG DT+ mice, co‐cultured with 2 µg/mL ConA. (D) Representative images of CD40^+^ cells within CD11b^+^ APCs in spleen cells from both AE‐DEREG DT‐ and AE‐DEREG DT+ mice at 1 month post‐infection (non‐infected mice as Control mice), co‐cultured with 2 µg/mL ConA. The same cell reactions performed without ConA were used as non‐stimulated controls. DT application with 110 ng/in jection/mouse started 1 day before infection and was maintained for 4 months (three times/week). Data represent mean ± SD of three independent experiments of a total of 8–10 mice in each group (4–5 mice per group in each independent experiment). Comparison between groups was performed using a one‐way ANOVA with Bonferroni's multiple comparison post‐test for statistical analysis. *p < 0.01. “DEREG DT‐,” *foxp3 inducible* knock‐down mice (DEREG mice) without DT application; “DEREG DT+,” DEREG mice with DT application; “AE‐ DEREG DT‐,” *E. multilocularis*‐infected DEREG without DT application; “AE‐ DEREG DT+,” *E. multilocularis*‐infected DEREG mice with DT application. “Control,” non‐infected mice; “1 m,” 1‐month p.i.; “4 m,” 4 months p.i.. “PEC,” peritoneal exudate cells; “Spleen,” spleen cells.

The role of FoxP3 in the co‐stimulation of CD11b^+^ and CD11c^+^ APCs was measured, in both PECs and spleen cells from AE‐DEREG DT+, AE‐DEREG DT‐ mice, and their non‐infected controls, respectively. Among CD11b^+^ APCs, the frequency of CD40 and MHCII was higher at 1‐month p.i. in spleen cells but not in PECs of AE‐DEREG DT+, when compared to AE‐DEREG DT‐ mice (Fig. 2A and B). However, among CD11c^+^ APCs there was no difference in the MHCII frequency between AE‐DEREG DT+ mice and AE‐DEREG DT‐ mice (data not shown).

Subsequently, we assessed the same parameters but upon in vitro cultivation and stimulation with ConA. Findings revealed that CD40 frequency in CD11b^+^ APCs from AE‐DEREG DT+ at 1‐month p.i. was significantly higher than in AE‐DEREG DT‐ mice (Fig. 2C and D). MHCII frequency yielded no difference in both subpopulations of CD11b^+^ and CD11c^+^ APCs.

### Co‐stimulatory signals for T cell activation and survival are less affected in DEREG DT+ than in DEREG DT‐ mice at the early stage of infection (1 month p.i.)

**Figure 3:**
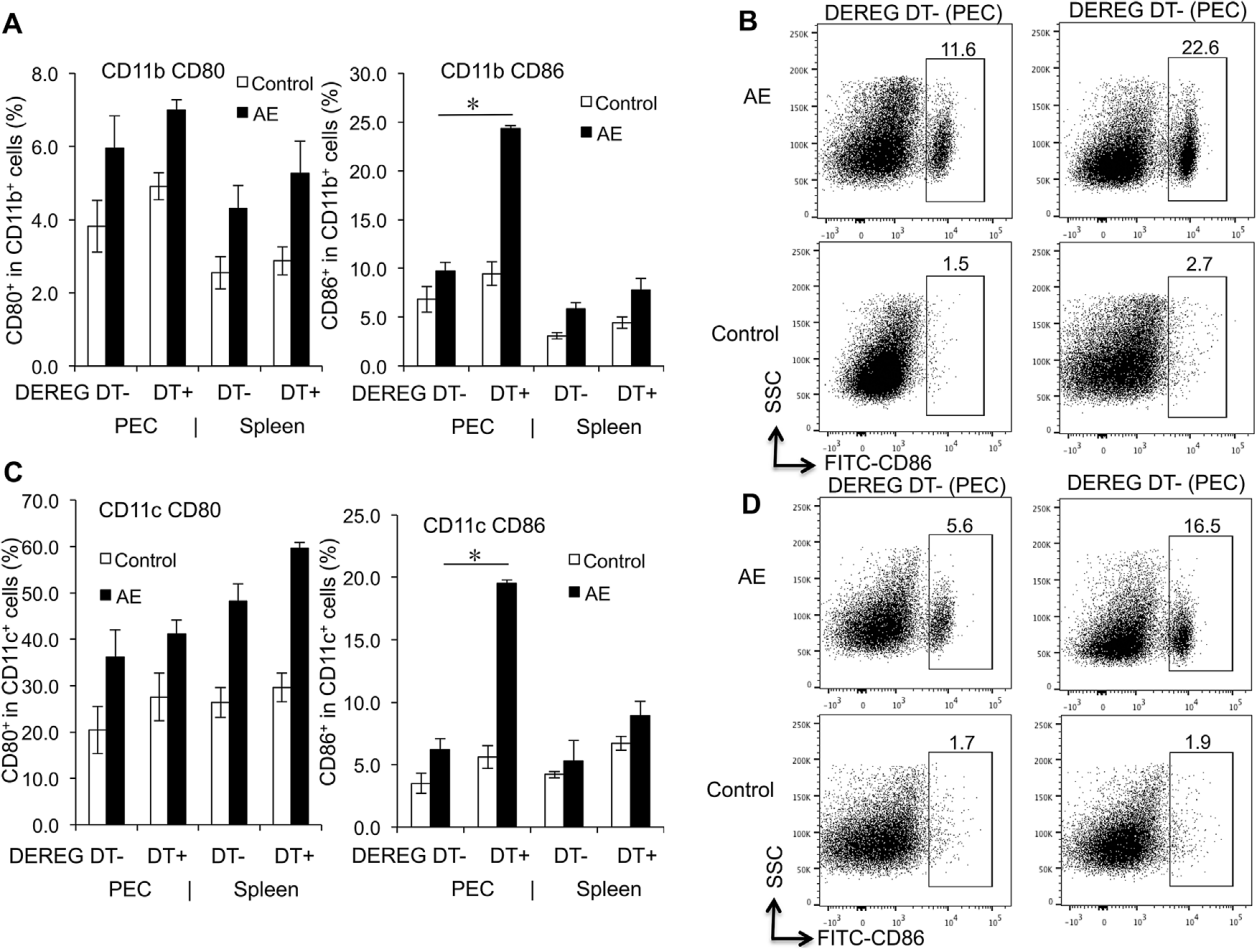
CD80 and CD86 expression levels in both CD11b^+^ and CD11c^+^ APCs, in both AE‐DEREG DT‐ and AE‐DEREG DT+ mice at 1 month post‐infection. (A) Frequency of CD80 and CD86 within CD11b^+^ APCs in peritoneal and spleen cells from AE‐DEREG DT‐ and AE‐DEREG DT+ mice 1 month post infection. (B) Representative images of CD86^+^ cells within CD11b^+^ cells in PECs from both AE‐DEREG DT‐ and AE‐DEREG DT+ mice at 1 month post‐infection, non‐infected mice as Control mice. (C) Frequency of CD80 and CD86 within CD11c^+^ APCs in peritoneal and spleen cells from AE‐DEREG DT‐ and AE‐DEREG DT+ mice 1 month post infection. (D) Representative images of CD86^+^ cells within CD11c^+^ APCs in PECs from both AE‐DEREG DT‐ and AE‐DEREG DT+ mice at 1 month post‐infection, non‐infected mice as Control mice. DT application with 110 ng/injection/mouse started 1 day before infection and was maintained for 4 months (three times/week). Data represent mean ± SD of three independent experiments of a total of 8–10 mice in each group (4–5 mice per group in each independent experiment). Comparison between groups was performed using a one‐way ANOVA with Bonferroni's multiple comparison post‐test for statistical analysis. *p < 0.01. “DEREG DT‐,” *foxp3 inducible* knock‐down mice (DEREG mice) without DT application; “DEREG DT + ”, DEREG mice with DT application; “AE‐ DEREG DT‐,” *E. multilocularis*‐infected DEREG without DT application; “AE‐ DEREG DT+,” *E. multilocularis*‐infected DEREG mice with DT application. “Control,” non‐infected mice; “1 m,” 1‐month p.i.; “4 m,” 4 months p.i.. “PEC,” peritoneal exudate cells; “Spleen,” spleen cells.

Anticipating a putative effect of FoxP3 on APCs for T cell activation and survival during *E. multilocularis* infection, we investigated co‐stimulatory markers for T cell activation and survival CD80 and CD86 in CD11b^+^ and CD11c^+^ APCs, in both PECs and spleen cells from AE‐DEREG DT+, and AE‐DEREG DT‐ mice and respective non‐infected controls. Flow cytometry showed that, in both CD11b^+^ and CD11c^+^ APCs, the frequency of the maturation marker CD86 in PECs but not in spleen cells was higher in AE‐DEREG DT+ than in AE‐DEREG DT‐ mice (Fig. 3A–D). However, in both CD11b^+^ and CD11c^+^ APCs, there was no difference in CD80 frequency between AE‐DEREG DT+ and AT‐DEREG DT‐ mice (Fig. 3A and C).

**Figure 4:**
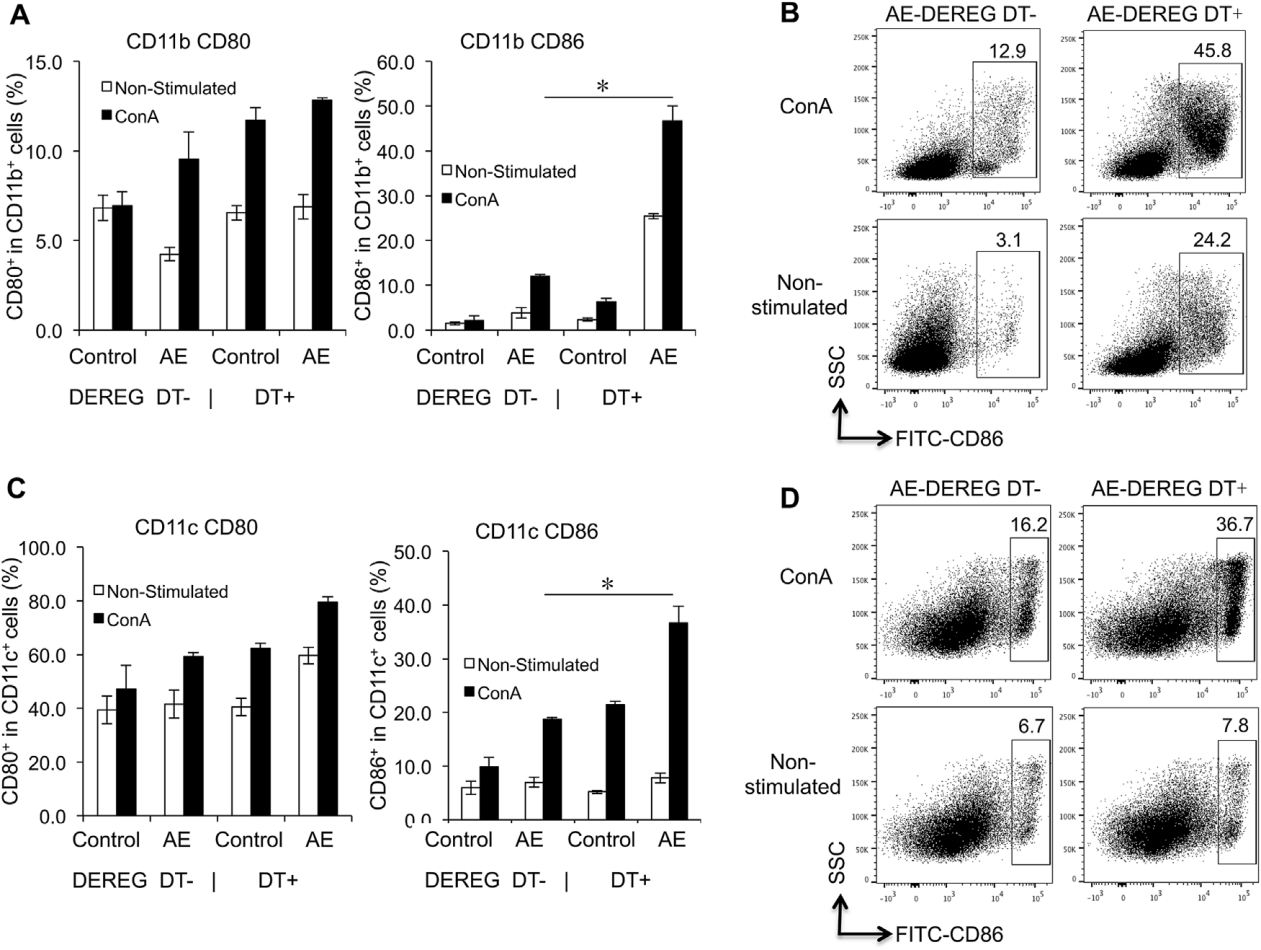
CD80 and CD86 expression levels in both CD11b^+^ and CD11c^+^ APCs, in response to ConA in both AE‐DEREG DT‐ and AE‐DEREG DT+ mice at 1 month post‐infection. (A) Frequency of CD80 and CD86 within CD11b^+^ APCs in spleen cells from AE‐DEREG DT‐ and AE‐DEREG DT+ mice 1 month post infection, co‐cultured with 2 µg/mL ConA. (B) Representative images of CD86^+^ cells within CD11b^+^ APCs in spleen cells from both AE‐DEREG DT‐ and AE‐DEREG DT+ mice at 1 month post‐infection (non‐infected mice as Control mice), co‐cultured with 2 µg/mL ConA. (C) Frequency of CD80 and CD86 within CD11c^+^ APCs in spleen cells from AE‐DEREG DT‐ and AE‐DEREG DT+ mice 1 month post infection, co‐cultured with 2 µg/mL ConA. (D) Representative images of CD86^+^ cells within CD11c^+^ APCs in spleen cells from both AE‐DEREG DT‐ and AE‐DEREG DT+ mice at 1 month post‐infection (non‐infected mice as Control mice), co‐cultured with 2 µg/mL ConA. The same cell reactions performed without ConA were used as non‐stimulated controls. DT application with 110 ng/injection/mouse started 1 day before infection and was maintained for 4 months (three times/week). Data represent mean ± SD of three independent experiments of a total of 8–10 mice in each group (4–5 mice per group in each independent experiment). Comparison between groups was performed using a one‐way ANOVA with Bonferroni's multiple comparison post‐test for statistical analysis. *p < 0.01. “DEREG DT‐,” *foxp3 inducible* knock‐down mice (DEREG mice) without DT application; “DEREG DT+,” DEREG mice with DT application; “AE‐ DEREG DT‐,” *E. multilocularis*‐infected DEREG without DT application; “AE‐ DEREG DT+,” *E. multilocularis*‐infected DEREG mice with DT application. “Control,” non‐infected mice; “1 m,” 1‐month p.i.; “4 m,” 4 months p.i.

Subsequently, we assessed the same parameters but upon in vitro cultivation and stimulation with ConA. Findings revealed that CD86 frequency in both CD11b^+^ and CD11c^+^ APCs from AE‐DEREG DT+ at 1‐month p.i. was significantly higher than in those from AE‐DEREG DT‐ mice, while CD80 frequency yielded no difference in both CD11b^+^ and CD11c^+^ APCs (Fig. 4A–D).

### Th1/Th17 immune response is enhanced in FoxP3 when compared to WT mice at the early stage of infection (1 month p.i.)

**Figure 5:**
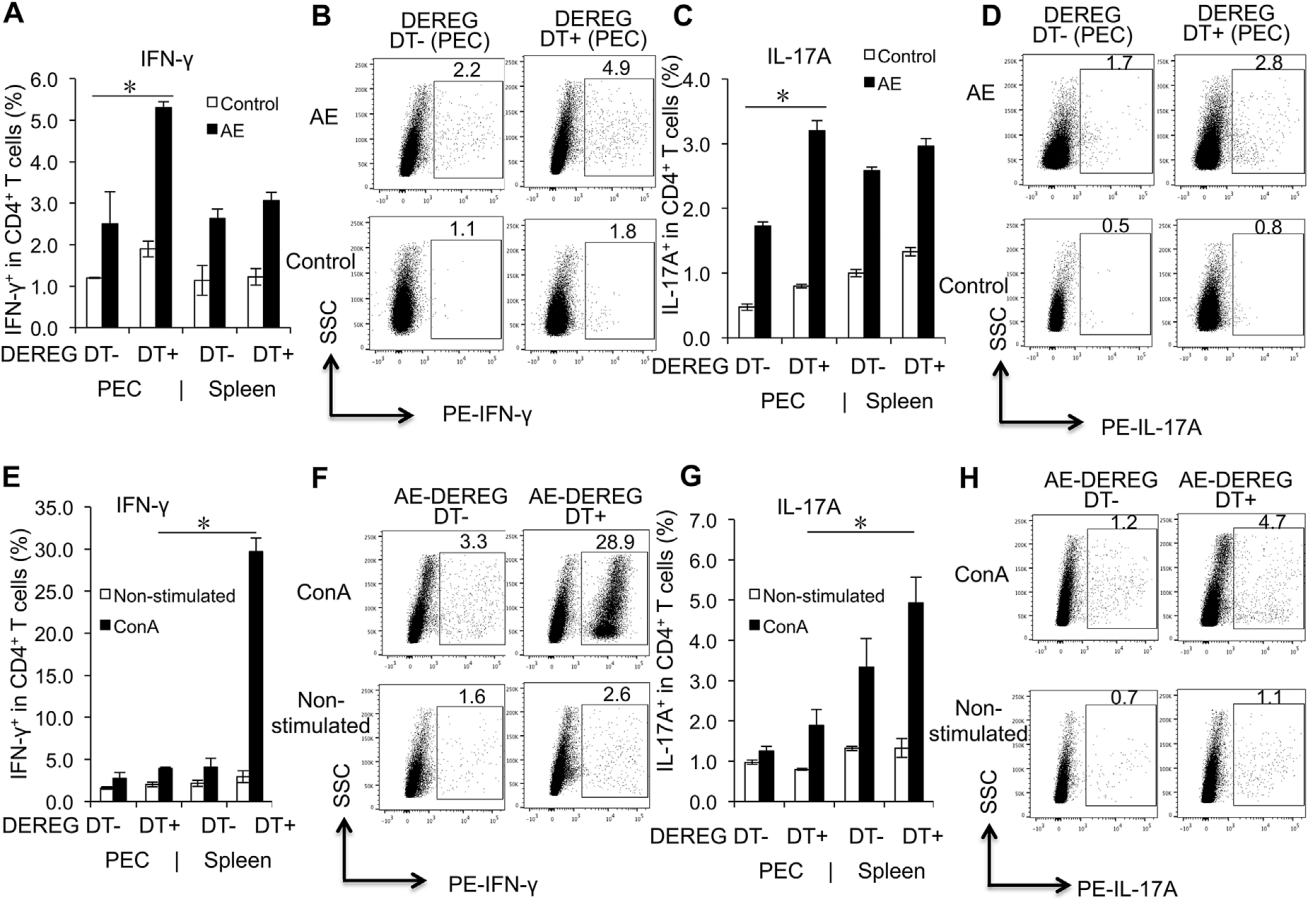
Th1/Th17 cell related cytokine expression in both AE‐DEREG DT‐ and AE‐DEREG DT+ mice at 1 month post‐infection, and in response to ConA. (A) Frequency of IFN‐γ^+^ within CD4^+^ T cells in peritoneal and spleen cells from AE‐DEREG DT‐ and AE‐DEREG DT+ mice at 1 month post infection, non‐infected mice as control mice. (B) Representative images of IFN‐γ^+^ cells within CD4^+^ T cells in PECs from both AE‐DEREG DT‐ and AE‐DEREG DT+ mice at 1 month post‐infection, non‐infected mice as control mice. (C) Frequency of IL‐17A^+^ within CD4^+^ T cells in peritoneal and spleen cells from AE‐DEREG DT‐ and AE‐DEREG DT+ mice at 1 month post infection, non‐infected mice as control mice. (D) Representative images of IL‐17A^+^ cells within CD4^+^ T cells in PECs from both AE‐DEREG DT‐ and AE‐DEREG DT+ mice at 1 month post‐infection, non‐infected mice as control mice. (E) Frequency of IFN‐γ^+^ within CD4^+^ T cells in peritoneal and spleen cells from AE‐DEREG DT‐ and AE‐DEREG DT+ mice (non‐infected mice as Control mice), co‐cultured with 2 µg/mL ConA. (F) Representative images of IFN‐γ^+^ cells within CD4^+^ T cells in spleen cells from both AE‐DEREG DT‐ and AE‐DEREG DT+ mice at 1 month post‐infection (non‐infected mice as Control mice), co‐cultured with 2 µg/mL ConA. (G) Frequency of IL‐17A^+^ within CD4^+^ T cells in peritoneal and spleen cells fromAE‐DEREG DT‐ and AE‐DEREG DT+ mice (non‐infected mice as Control mice), co‐cultured with 2 µg/mL ConA. (H) Representative images of IL‐17A^+^ cells within CD4^+^ T cells in spleen cells from both AE‐DEREG DT‐ and AE‐DEREG DT+ mice at 1 month post‐infection (non‐infected mice as Control mice), co‐cultured with 2 µg/mL ConA. The same cell reactions performed without ConA were used as non‐stimulated controls. DT application with 110 ng/injection/mouse started 1 day before infection and was maintained for 4 months (three times/week). Data represent mean ± SD of three independent experiments of a total of 8–10 mice in each group (4–5 mice per group in each independent experiment). Comparison between groups was performed using a one‐way ANOVA with Bonferroni's multiple comparison post‐test for statistical analysis. *p < 0.0125. “DEREG DT‐,” *foxp3 inducible* knock‐down mice (DEREG mice) without DT application; “DEREG DT+,” DEREG mice with DT application; “AE‐ DEREG DT‐,” *E. multilocularis*‐infected DEREG without DT application; “AE‐ DEREG DT+,” *E. multilocularis*‐infected DEREG mice with DT application. “Control,” non‐infected mice; “1 m,” 1‐month p.i.; “4 m,” 4 months p.i.. “PEC,” peritoneal exudate cells; “Spleen,” spleen cells.

To further explore the effect of FoxP3 on the immune response during *E. multilocularis* infection, firstly, Th‐related cytokines were comparatively assessed in AE‐DEREG DT+ mice, in AE‐WT mice, and in their respective non‐infected controls. Flow cytometry revealed that T helper (Th) cells from AE‐DEREG DT+ mice were oriented toward a Th1 and Th17 pathway in PECs of mice at early stage of infection (1 month p.i.). Respective flow cytometric analyses showed that there was no difference between spleen cells from AE‐DEREG DT+ and AE‐DEREG DT‐ regarding expression of IFN‐γ^+^ and IL‐17A^+^ within CD4^+^ T cells (Fig. 5A–D). Subsequently, we assessed the same parameters but upon in vitro cultivation and stimulation with ConA. We found that expression of IFN‐γ^+^ and IL‐17A^+^ within CD4^+^ T cells was significantly higher in spleen cells from AE‐DEREG DT+ at 1‐month p.i., 48 h after exposure to ConA, when compared to AE‐DEREG DT‐ mice (Fig. 5E–H).

### FoxP3 as a potential target for AE immunotherapy?

**Figure 6:**
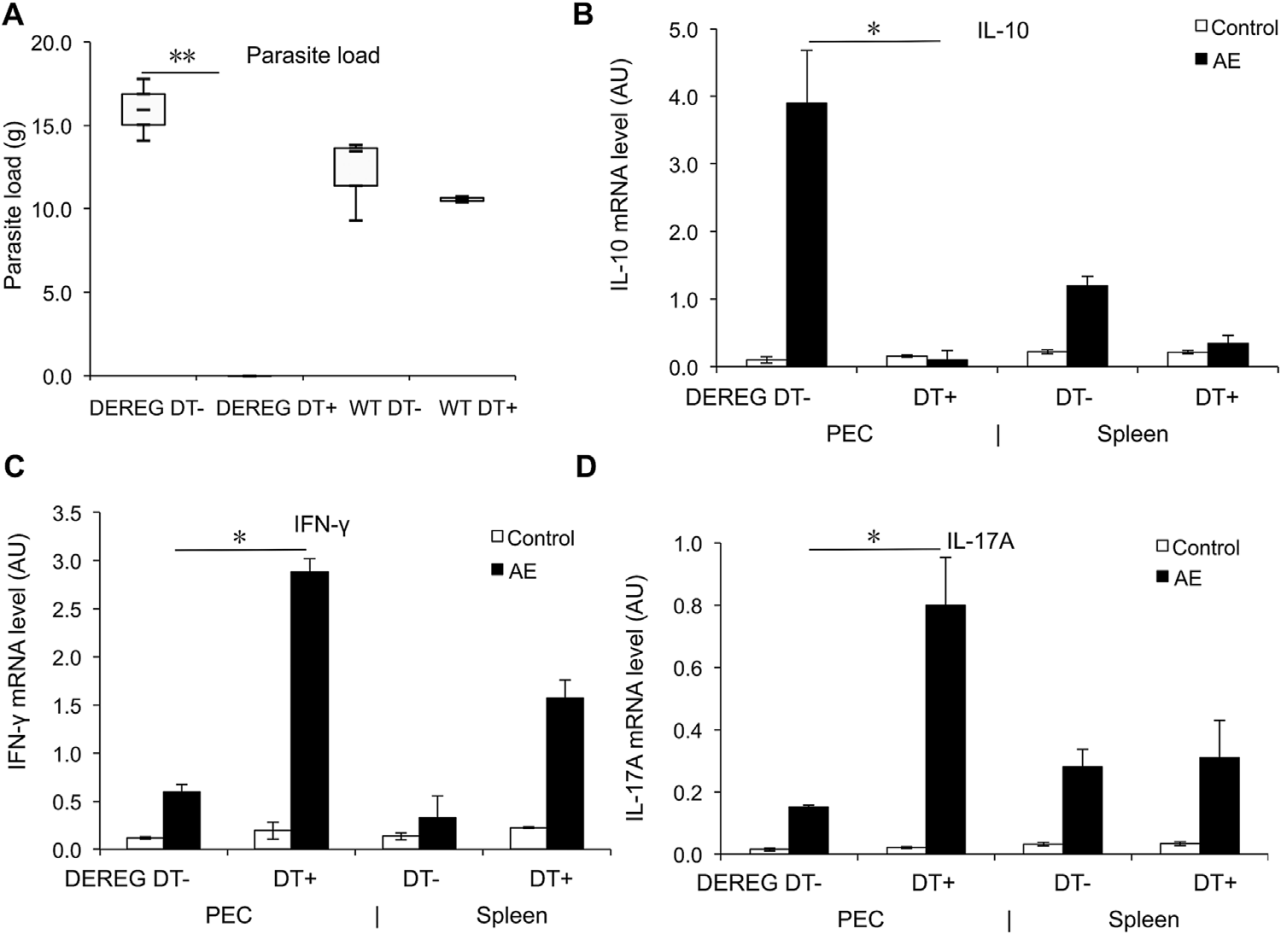
Effects of FoxP3 as a post‐infection treatment target, assessed upon parasite load in *E. multilocularis* infected mice, and related T cell cytokine expression measured by qRT‐PCR. (A) Parasite load in AE‐DEREG DT‐ and AE‐DEREG DT+ mice assessed by wet weight measurement at 4 months post‐infection. DT application with 110 ng/mouse (three times/week) started 6 weeks post infection and was maintained until 4 months. (B) *Il‐10* gene expression level in peritoneal and spleen cells from AE‐DEREG DT‐ and AE‐DEREG DT+ mice at 4 months post infection. (C) *Ifn‐γ* gene expression level in peritoneal and spleen cells from AE‐DEREG DT‐ and AE‐DEREG DT+ mice at 4 months post infection. (D) *Il‐17a* gene expression level in peritoneal and spleen cells from AE‐DEREG DT‐ and AE‐DEREG DT+ mice at 4 months post infection. Data represent mean ± SD of two independent experiments of a total of 8–10 mice in each group (4–5 mice per group in each independent experiment). Comparison between groups was performed using a one‐way ANOVA with Bonferroni's multiple comparison post‐test for statistical analysis. *p < 0.017. “WT,” wild type; “DEREG DT‐,” *foxp3 inducible* knock‐down mice (DEREG mice) without DT application; “DEREG DT+,” DEREG mice with DT application; “AE‐WT,” *E. multilocularis*‐infected wild type mice; “AE‐DEREG DT+,” *E. multilocularis*‐infected DEREG mice with DT application. “Control,” non‐infected mice; “1 m,” 1 month p.i.; “4 m,” 4 months p.i.. AU: arbitrary units.

To explore whether FoxP3 could be tackled as a putative target of immunotherapy against chronic *E. multilocularis* infection, female‐10‐week‐old DEREG mice were intra‐peritoneally infected with *E. multilocularis*, and subsequently treated with DT for 3 months after the infection was established, that is, at 6 weeks p.i.. DT‐Treated mice (AE‐DEREG DT+) were compared to non‐treated mice (AE‐DEREG DT‐). We found that AE‐DEREG DT+ mice had no parasite growing at all when compared to AE‐DEREG DT‐ mice (0.00 ± 0.00 vs. 15.95 ± 2.61 g, *p *< 0.001 (Fig. 6A), and this effect was associated with a stronger Th1/Th17 response, and a lower IL‐10 expression level (Fig. 6B–D). In the AE‐DEREG DT+ mice, where no metacestode mass could be recovered post‐mortem, the initial infection efficacy was confirmed by appropriate seropositivity upon Em2‐ and Em18‐ELISA 9. To assess a potential non‐Treg related effect of DT application on the parasite growth in WT mice, we carried out the following experiment in parallel: DT (110 ng/mouse three times/week) was administered for a period of 3 months in wild‐type mice, beginning 6 weeks after *E. multilocularis* infection. There was no difference in parasite load between 3‐months‐DT‐treated and non‐treated normal AE‐WT mice (Fig. 6A).

## Discussion

Chronic infection with *E. multilocularis* is characterized by a marked immune tolerance status, which mainly includes a down‐regulation of periparasitic effector mechanisms; this down‐regulation of host immune response develops progressively and reaches its maximum at the late stage of infection/disease, both in human patients 10 and in experimentally infected mice 11. In the present study, we identified FoxP3‐expressing Tregs as an important immunological factor promoting *E. multilocularis* proliferative activity in mice. We showed that FoxP3‐expressing Tregs (i) contributed to suppressing Th1‐ and Th17‐type immune responses and (ii) down‐regulated APC co‐stimulatory activities. Furthermore, we showed that down‐regulation of FoxP3‐expression had the potential to be exploited for the development of an immunotherapeutic strategy to reinforce the control of larval *E. multilocularis‐*infection by interfering with the immunoregulatory pathways promoted by the parasite in its host.

Our earlier studies had shown that CD4^+^CD25^+^ Tregs were up‐regulated in PECs from wild‐type *E. multilocularis*‐infected mice 8 when compared to non‐infected littermates. Since Tregs appeared as one of the key immune subsets to mediate the anergic immune status observed in chronically infected mice, our present objective was to study the role of FoxP3 Tregs in the co‐modulation of the outcome of *E. multilocularis* infection. Infecting DEREG mice with *E. multilocularis* metacestode offered us a unique tool to compare *E. multilocularis* hosts with and without depletion of inducible FoxP3. This allowed us to demonstrate that metacestode growth in AE‐DEREG mice after depletion of inducible FoxP3 Tregs yielded a significantly lower parasite load not only when DT was administered preventively before *E. multilocularis* infection, but also when DT was administered therapeutically when the infection was already established. The latter observation is really unique if we consider all attempts to modulate the immune response reported previously 12, since it resulted in a complete disappearance of liver lesions in infected DEREG mice treated by DT after 6 weeks of infection. The effect on metacestode growth was actually related to FoxP3+Treg depletion since DT added to metacestode culture did not kill the parasite in vitro, and wild‐type mice treated by DT did not exhibit any changes in parasite load. We were also able to show that prevention and arrest of *E. multilocularis* growth in DT‐treated DEREG mice was associated with down‐regulation of T cell function, of T‐cell co‐stimulation, and of dendritic cell (DC) maturation. Influence of regulatory T cell depletion on the outcome of infection has been shown in several models. Littwitz‐Salomon et al. 13 showed a strong increase of NK cell maturation, activation and effector functions in Treg‐depleted mice, and activated regulatory T cells suppressed effector NK cell responses by an IL‐2‐mediated mechanism during an acute retroviral infection. Another study showed a significantly increased IL‐6 level and a trend toward higher levels of TNF‐α in Treg‐depleted mice during sepsis, which supported the hypothesis that the hyper‐inflammatory response during the early phase of sepsis was intensified in mice lacking Treg cells. Regarding parasitic infections, Treg cell depletion was shown to augment a vaccine‐induced T effector cell response against the liver‐stage of malaria even though it failed to increase memory 14. With regard to malignant tumors, that share many immunological characteristics with parasites, Klages et al. 15 showed that Foxp3^+^ Treg depletion at an intermediate time point resulted in an efficient regression of melanoma tumor growth, and very efficiently controlled the homeostasis of CD8^+^ T cells especially within the tumor site. Furthermore, combining selective Foxp3^+^ Treg depletion with vaccination improved the regression of established tumors 15. Taken together, our results confirm those obtained with other models of infection or cancer associated with immune evasion mechanisms and support the hypothesis that the parasite's strategy to increase its survival and growth potential focuses on the involvement of FoxP3‐expressing Tregs, which contributes to the development of a tolerance immune status in chronically infected mice. Several studies in experimental mice and in patients with AE suggested that in chronic infection effector mechanisms were at least partially inhibited despite the presence of potentially cytotoxic cells 3, 12, 16. In the present study, we showed that depletion of Foxp3^+^‐Tregs in DEREG‐mice was accompanied by a higher costimulatory activity in both CD11b^+^ and CD11c^+^ APCs and by a stronger Th1/Th17 immune response leading to an improved control of *E. multilocularis* infection. Thus in normal experimental hosts, Foxp3^+^‐Tregs, specifically induced by secretory/excretory products of the metacestode, as suggested by our in vitro experiments, might prevent APC activation, limit the signal strength to T cell priming, and permanently decrease Th1/Th17 effector response while enhancing Th2‐type cytokines, a hallmark of the immune profile of AE 3, 12.

One striking observation was the lack of difference in resistance between *E. multilocularis*‐infected DEREG mice that were DT‐treated for only 1 month versus those treated for 4 months; both groups exhibited similarly reduced parasite burden. In a recently published paper, we showed that Foxp3 could not be kept knocked‐down for a longer period of DT administration due to subsequent antibody formation by treated animals and DT‐neutralization via DT‐specific antibodies 17. To explain the mechanism that ensures that the Treg niche is rapidly refilled following depletion, Nyström et al. 18 suggested that these Treg cells could be (i) the product of peripheral conversion of activated conventional cells to peripherally induce Treg (pTreg) cells or (ii) homeostatic proliferation of existing thymus‐derived Treg (tTreg) cells. A separate study found that homeostatic expansion of Treg cells (through proliferation and reduced apoptosis) could completely restore the compartment following 50% Treg‐cell depletion, with no evidence of conversion of conventional T cells observed 19. An additional parameter contributing to this phenomenon may be by neutralizing‐.antibodies that may affect DT‐function concerning inhibition of Foxp3‐expression 17.

How FoxP3^+^ Tregs causatively influence and/or modulate the outcome of experimental AE is still unclear, although various molecular and cellular mechanisms have been proposed to explain how Tregs may basically suppress immune responses. These include cell‐to‐cell contact‐dependent suppression, cytotoxicity, and immunoregulatory cytokine secretion such as IL‐10 and TGF‐β 20, two immunoregulatory cytokines widely expressed in the liver lesions and present in the plasma of patients with AE 4, 7, 12. However, the importance of these cytokines remains controversial, as several studies have demonstrated that antibodies directed against IL‐10 and TGF‐β failed to block Treg suppressive function, and Tregs from TGF‐β–deficient mice retained normal suppressive activity in vitro 20. In addition, the ambiguous role of TGF‐β, which is both a strong inducer of immune tolerance and an activator of the pro‐inflammatory IL‐17 cytokine system, remains puzzling 21, 22. However, previous studies strongly suggest that both regulatory cytokines are important actors in *Echinococcus* spp. immune evasion 5, 7, 10. Specific influence of parasitic products on IL‐23 could be a key‐step in the modulation of IL‐17 promotion or suppression and of Th17/Treg balance 5, and should be studied more in depth in future work.

Our findings on the role of Foxp3^+^ Tregs in *E. multilocularis* infection now open the door for more applied approaches. Understanding the mechanism by which FoxP3 expression regulates the immune process in AE could help find new immunotherapeutic targets, since the metacestode disappearance we observed by depleting FoxP3 Treg cells in hosts with fully established metacestode was never equaled with other types of immune manipulation; up to now, only pre‐infection administration of IL‐12 was able to effectively suppress AE occurrence in the mouse model; as this was not applicable to humans, it was never tested as a therapeutic agent 23. However, directly targeting FoxP3 and acting on all FoxP3‐expressing Treg cells without discrimination might be harmful, especially by promoting allergy and auto‐immunity. Treg cells play a role in the development of the immune response after birth and have been associated with environmental conditions which promote protection against allergy; however, this positive influence of Tregs to prevent allergy is operating in early life, and is far less crucial later on 24. Contribution of Tregs to alleviation of experimental allergic asthma has also been shown after specific immunotherapy 25; however, a direct contribution of Tregs to the development of IgE‐dependent allergy in adults is more controversial. Treg cells play a dominant role in the suppression of autoimmune reactions/diseases. In the mouse, a lack of CD4^+^ FoxP3^+^ Tregs resulted in increased autoimmunity, and adoptive transfer of Treg prevented and reversed autoimmunity 26. In humans, lack of functional Tregs also leads to autoimmunity, as is seen in individuals who have immune‐dysregulative polyendocrinopathy and enteropathy X‐linked (IPEX) syndrome due to mutations in FoxP3. These individuals develop aggressive autoimmunity including insulin‐dependent diabetes, thyroiditis and eczema 27, 28. Depletion of Tregs also increased disease severity of actively induced and adoptively transferred murine autoimmune neuritis 29. However, Savarin et al. 30 also showed that residual Foxp3^+^ Tregs detected 1 week after DT treatment might be sufficient to control central nervous system (CNS) inflammation. The minor Foxp3^+^ Treg population detected at day 28 p.i. presumably comprised rebound Tregs, as well as a small proportion of DT‐insensitive Foxp3^+^ cells detected in adult DEREG mice 31. While rebound Tregs are dysfunctional 32, DT‐resistant Foxp3^+^ Tregs have been shown to protect against lethal autoimmunity and suffice to control the self‐reactive (SR) CD4^+^ T cells during chronic mouse hepatitis virus infection 31. Our observations made in AE‐DEREG mice DT‐treated for only 1 month versus those treated for 4 months might support an efficient action on parasite growth using time‐limited administration of anti‐Treg biological agents while avoiding auto‐immune reactions. All these issues should be studied very carefully before any therapeutic application to humans, keeping in mind that AE is a very severe tumor‐like disease still without fully effective anti‐infective therapy 1.

## Materials and Methods

### Ethics statement

The animal study was performed in strict accordance with the recommendations of the Swiss Guidelines for the Care and Use of Laboratory Animals. The protocol was approved by the governmental Commission for Animal Experimentation of the Canton of Bern (approvals no. BE103/11 and BE112/14).

### Experimental design, parasite sampling, and histological examinations

#### Mice

DEREG (depletion of regulatory T cells) mice of the C57/BL6‐background and respective wild‐type littermates, all aged between 8 and 10 weeks, were used for intraperitoneal infection with *E. multilocularis* as previously described 33. Age‐and‐gender‐matched littermates were used for mock‐infected control groups. In DEREG mice, a diphtheria toxin (DT) receptor together with an enhanced green fluorescent protein (eGFP) is expressed under the control of the FoxP3 promoter. Therefore, depletion of Treg cells can be achieved by appropriate DT application 32. As shown elsewhere 34, in naïve DEREG mice a subset of FoxP3^+^ Treg cells lacking the DT receptor and GFP expression is not affected by the treatment. All animals were bred and housed under specific pathogen free (SPF) conditions according to recommendations of the Federation of European Laboratory Animal Science Association (FELASA), and additionally monitored by daily inspection, including the assessment of the appearance of health status, putative weight loss or gain during the whole course of the experiment. All experiments with animals were performed within a laminar flow safety enclosure.

#### Parasite and experimental infection

*E. multilocularis* (H95) was isolated and maintained by serial passages (vegetative transfer) in C57BL/6 mice as previously described 35. In order to prepare the infection material for mice, metacestode tissue was obtained from previously infected mice by aseptic removal from the peritoneal cavity. After grinding the tissue through a sterile 50 µm sieve, approximately 100 freshly prepared vesicular cysts were suspended in 100 µL RPMI‐1640 (Gibco, Basel, Switzerland) and intraperitoneally (i.p.) injected. Each experimental group included six animals unless otherwise stated. Control mice (mock‐infection) received 100 µL of RPMI‐1640 only.

#### Treg cell depletion

DEREG‐C57/BL6 mice (DEREG) and their WT littermate (WT) controls were either used as infected animals or as non‐infected controls. All mice belonging to the Treg cell depletion group (knockouts and WT control animals) received 110 ng of DT i.p. (Calbiochem, Merck, Germany) dissolved in 100 μl PBS at 1 day before infection till (a) 1 month p.i. with a frequency of three applications/week, or until (b) 4 month p.i., with a frequency of three applications/week. Successful depletion and rebound of Treg cells was confirmed by flow cytometric analysis based on the detection of the FoxP3eGFP signal in splenic leukocytes. Infected mice were daily monitored for survival and morbidity.

#### Parasite tissue mass and quantification

Mice were sacrificed by CO_2_‐euthanasia at 1 month (corresponding to an early stage of chronic infection) and 4 months (corresponding to a middle/late = chronic stage of disease) p.i.. Blood was collected by cardiac puncture, and serum samples were stored at −80°C. Parasite tissues were dissected and, if present, fat and connective tissues were carefully removed for subsequent determination of the parasite mass.

#### Cell preparations

Peritoneal exudate cells (PEC) and spleen cells from naïve (control) and *E. multilocularis*‐infected (AE) DEREG and WT mice were collected by peritoneal rinsing, or grinding separately with 5 mL RPMI‐1640. Cells were subsequently washed twice and resuspended in RPMI‐1640 (Gibco) for cell staining or cell culture. Macrophages were removed from each group of mice by plastic‐adhesion after incubation of PEC or spleen cell suspension in 15 mL RPMI‐1640 + 20% FCS in a petri‐dish for 2 h at 37°C, in an atmosphere containing 5% CO_2_. Subsequent to incubation, non‐adherent cells were separated from macrophage‐enriched adherent cells, and this new cell suspension was used for FACS analyses.

### Spleen cell stimulation assays

Spleen cells were cultured at a density of 2 × 10^6^ cells/mL in RPMI‐1640 +10%FCS, and stimulated with 2 µg/mL ConA for 48 h, or with different concentrations of VF for 96 h, aimed at surface marker staining. The same cell reactions performed without ConA or VF were used as negative controls. The same stimulation but in the presence of protein‐transport inhibitor‐cocktail was used for cytokine staining.

### Flow cytometry

Aliquots of 10^6^ cells/100 µL of staining buffer per well were incubated each with 1 µg of purified anti‐CD16/CD32 for 20 min in the dark, in order to block non‐specific binding of antibodies to the FcγIII and FcγII receptors. Subsequently, these cells were separately stained with the following surface markers for 15 min with 1 µg of primary antibodies: APC‐labeled anti‐CD4, FITC‐labeled anti‐CD25, anti‐CD80, anti‐CD86, anti‐CD40, anti‐MHCII; PE‐labeled anti‐CD11b, anti‐CD11c. All antibodies were from eBioscience (San Diego, CA). For intracellular staining, PECs or spleen cells were first incubated with Inside Fix (Miltenyi Biotec, Bergisch Gladbach, Germany) for 20 min at room temperature and subsequently stained with PE‐labeled anti‐IFN‐γ, anti‐IL‐4, anti‐IL‐17A, anti‐IL‐10 and anti‐Foxp3 (eBioscience, San Diego, CA) in Inside Perm (Miltenyi Biotec, Bergisch Gladbach, Germany) for 15 min in the dark. Corresponding fluorochrome‐labeled isotype control antibodies were used for staining controls. Cells resuspended in 300 µL of buffer (0.15 M NaCl, 1 mM NaH_2_PO_4_ H_2_O, 10 mM Na_2_HPO_4_ 2H_2_O and 3 mM NaN_3_) were analyzed in a flow cytometer (Becton Dickinson, Heidelberg, Germany) using the corresponding CELL QUEST software.

### Total RNA extraction and qRT‐PCR

Total RNA was isolated from spleens using the Qiagen RNeasy MiniKit according to the manufacturer's instructions. The quality of the isolated RNA was determined with a NanoDrop ND 1000 (NanoDrop Technologies, Witec AG, Lucerne, Switzerland) and a Bioanalyzer 2100 (Agilent Technologies AG, Bassel, Switzerland). Only samples with a 260‐nm:280‐nm ratio between 1.9 and 2.1 and a 28S:18S ratio within 1.5 to 2 were further processed. cDNA was synthesized using the Omniscript Reverse Transcription kit (Qiagen, Hilden, Germany). SYBR‐Green Mix‐based qRT‐PCR was carried out on a Rotor‐Gene 6000 qPCR detection system (Corbett) with the FastStart Essential DNA Green Master (Roche, Basel, Switzerland) following the manufacturer's instructions. PCR cycling was performed in triplicates in final volumes of 20 µL containing 2 μL cDNA and 10 pM of each primer (Cycle scheme: initial denaturation at 95°C—15 min, 45 cycles of 95°C—15 s, 55°C − 30 s, and 72°C—30 s). Fluorescence was measured in every cycle, and a melting curve was analyzed after the PCR by increasing the temperature from 55 to 95°C in 0.5°C increments. The primers used were described earlier 4, and mRNA levels of different cytokines were quantified relative to the mRNA level of housekeeping gene *β‐actin*.

### Statistical analyses

All data were analyzed by SPSS 17.0. The results are presented as means ± SD. Normality of data was assessed by D'Agostino & Pearson and Shapiro–Willk test. For normally distributed groups pf data, One‐way ANOVA followed by Bonferroni's post‐test or un‐paired two‐tail Student's *t*‐test were used to compare the differences between groups. Significance was defined as *p *< 0.05 for all tests, except those subsequently corrected by Bonferroni.

## Conflicts of Interest

The authors declare no commercial or financial conflicts of interest.

## Supporting information

Additional supporting information may be found in the online version of this article at the publisher's web‐site.

**Figure S1**. Inhibition of FoxP3 expression with CD4 T cells in peritoneal cells from both AE‐DEREG DT‐ and AE‐DEREG DT+ mice at 1 month and 4 months post‐infection. (A) Frequency of FoxP3^+^ within CD4^+^ T cells in PECs from AE‐DEREG DT‐ and AE‐DEREG DT+ mice at 1 month and 4 months post‐infection, non‐infected mice as control mice, DT application with 110 ng/mouse (three times/week) started 1 day before infection and was maintained for 4 months. (B) Representative images of FoxP3^+^ within CD4^+^ T cells in PECs from AE‐DEREG DT‐ and AE‐DEREG DT+ mice at 1 month and 4 months post‐infection, non‐infected mice as control mice. DT application with 110 ng/mouse (three times/week) started 1 day before infection and was maintained for 4 months. (C) Frequency of FoxP3^+^ within CD4^+^ T cells in PECs from AE‐DEREG DT‐ and AE‐DEREG DT+ mice at 1 month and 4 months post‐infection, non‐infected mice as control mice. DT application with 110 ng/injection/mouse (three times/week) started 1 day before infection and was maintained for 1 month. (D) Representative images of FoxP3^+^ within CD4^+^ T cells in PECs from AE‐DEREG DT‐ and AE‐DEREG DT+ mice at 1 month and 4 months post‐infection, non‐infected mice as control mice. DT application with 110 ng/injection/mouse (three times/week) started 1 day before infection and was maintained for 1 month. Data represent mean ± SD of three independent experiments of a total of 8–10 mice in each group (4–5 mice per group in each independent experiment). Comparison between groups was performed using a one‐way ANOVA with Bonferroni's multiple comparison post‐test for statistical analysis. *p < 0.01. “DEREG DT‐,” *foxp3 inducible* knock‐down mice (DEREG mice) without DT application; “DEREG DT+,” DEREG mice with DT application; “AE‐DEREG DT‐,” *E. multilocularis*‐infected DEREG without DT application; “AE‐DEREG DT+,” *E. multilocularis*‐infected DEREG mice with DT application. “Control,” non‐infected mice; “1 m,” 1‐month p.i.; “4 m,” 4 months p.i.
