# Supplementary figures and images for: Depletion of FoxP3+ Tregs improves control of larval Echinococcus multilocularis infection by promoting co‐stimulation and Th1/17 immunity
Source: Immun Inflamm Dis. 2017 Jun 16;5(4):435–47. doi: 10.1002/iid3.181 (PMC5691311; doi:10.1002/iid3.181)

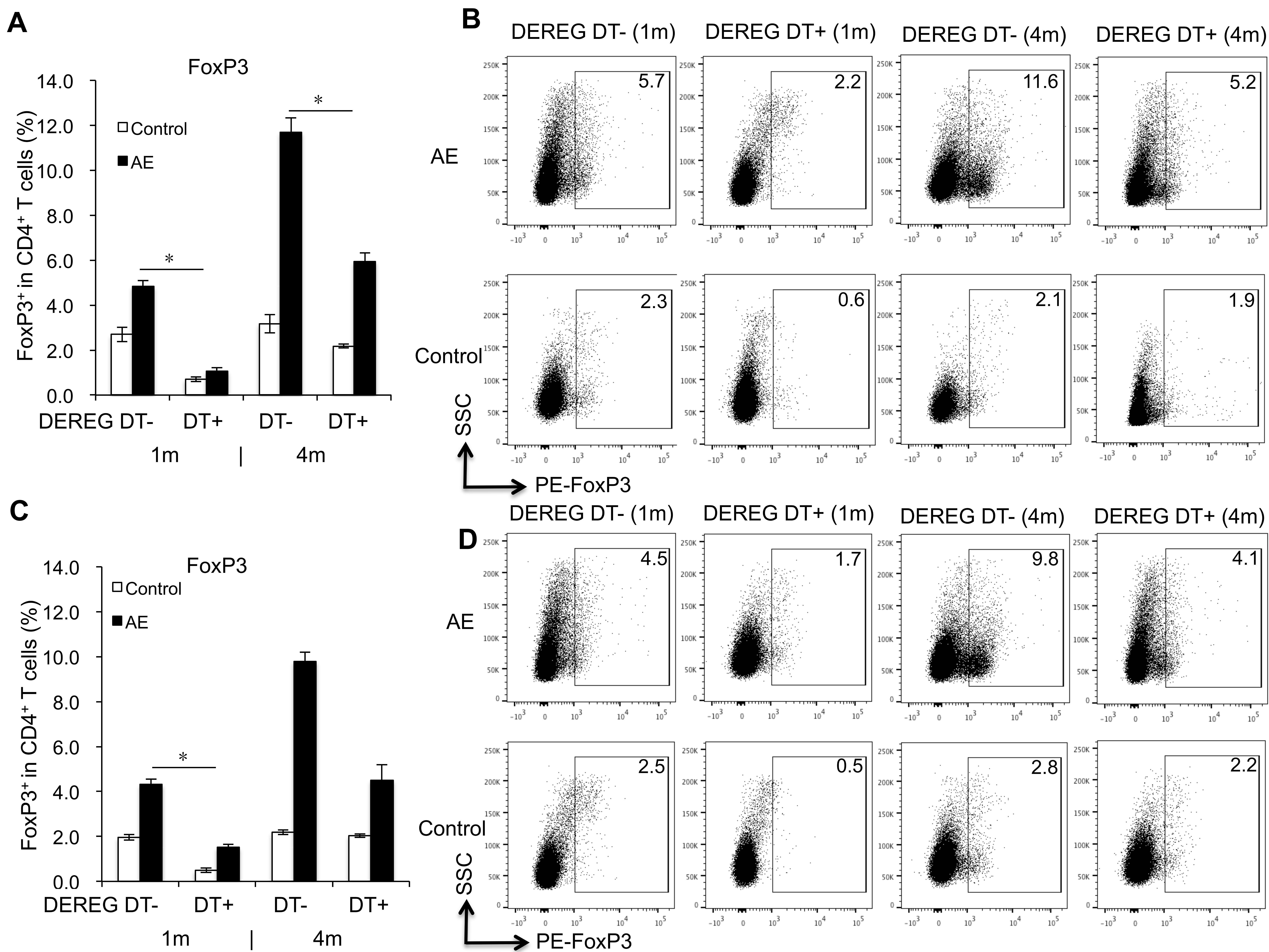

Supplement: Supplementary file 1 — Figure S1. Inhibition of FoxP3 expression with CD4 T cells in peritoneal cells from both AE‐DEREG DT‐ and AE‐DEREG DT+ mice at 1 month and 4 months post‐infection. (A) Frequency of FoxP3+ within CD4+ T cells in PECs from AE‐DEREG DT‐ and AE‐DEREG DT+ mice at 1 month and 4 months post‐infection, non‐infected mice as control mice, DT application with 110 ng/mouse (three times/week) started 1 day before infection and was maintained for 4 months. (B) Representative images of FoxP3+ within CD4+ T cells in PECs from AE‐DEREG DT‐ and AE‐DEREG DT+ mice at 1 month and 4 months post‐infection, non‐infected mice as control mice. DT application with 110 ng/mouse (three times/week) started 1 day before infection and was maintained for 4 months. (C) Frequency of FoxP3+ within CD4+ T cells in PECs from AE‐DEREG DT‐ and AE‐DEREG DT+ mice at 1 month and 4 months post‐infection, non‐infected mice as control mice. DT application with 110 ng/injection/mouse (three times/week) started 1 day before infection and was maintained for 1 month. (D) Representative images of FoxP3+ within CD4+ T cells in PECs from AE‐DEREG DT‐ and AE‐DEREG DT+ mice at 1 month and 4 months post‐infection, non‐infected mice as control mice. DT application with 110 ng/injection/mouse (three times/week) started 1 day before infection and was maintained for 1 month. Data represent mean ± SD of three independent experiments of a total of 8–10 mice in each group (4–5 mice per group in each independent experiment). Comparison between groups was performed using a one‐way ANOVA with Bonferroni's multiple comparison post‐test for statistical analysis. *p < 0.01. “DEREG DT‐,” foxp3 inducible knock‐down mice (DEREG mice) without DT application; “DEREG DT+,” DEREG mice with DT application; “AE‐DEREG DT‐,” E. multilocularis‐infected DEREG without DT application; “AE‐DEREG DT+,” E. multilocularis‐infected DEREG mice with DT application. “Control,” non‐infected mice; “1 m,” 1‐month p.i.; “4 m,” 4 months p.i. [file IID3-5-435-s001.tif]
